# GOBP1 Plays a Key Role in Sex Pheromones and Plant Volatiles Recognition in Yellow Peach Moth, *Conogethes punctiferalis* (Lepidoptera: Crambidae)

**DOI:** 10.3390/insects10090302

**Published:** 2019-09-17

**Authors:** Dapeng Jing, Tiantao Zhang, Shuxiong Bai, Sivaprasath Prabu, Kanglai He, Youssef Dewer, Zhenying Wang

**Affiliations:** 1 College of Plant Protection, Shenyang Agricultural University, Shenyang 110161, China; jingfly6@163.com; 2 State Key Laboratory for Biology of Plant Diseases and Insect Pests, Institute of Plant Protection, Chinese Academy of Agricultural Sciences, Beijing 100193, China; sxbai@ippcaas.cn (S.B.); sivaprasathibt@gmail.com (S.P.);; 3 Bioassay Research Department, Central Agricultural Pesticide Laboratory, Sabahia Plant Protection Research Station, Agricultural Research Center, Alexandria 21616, Egypt; dewer72@yahoo.com

**Keywords:** *Conogethes punctiferalis*, GOBPs, fluorescence competitive binding assays, circular dichroism, molecular docking

## Abstract

Insects recognize odorous compounds using sensory neurons organized in olfactory sensilla. The process odor detection in insects requires an ensemble of proteins, including odorant binding proteins, olfactory receptors, and odor degrading enzymes; each of them are encoded by multigene families. Most functional proteins seem to be broadly tuned, responding to multiple chemical compounds with different, but mostly quite similar structures. Based on the hypothesis that insects recognize host volatiles by means of general odorant binding proteins (GOBPs), the current study aimed to characterize GOBPs of the yellow peach moth, *Conogethes punctiferalis* (Guenée). In oviposition preference tests, it was found that the yellow peach moth preferred volatiles from *Prunus persica* (peach) in finding their host plant. Exposure of the moth to volatiles from peaches affected the expression level of GOBP genes. Binding affinity of GOBPs from yellow peach moth was assessed for 16 host plant volatiles and 2 sex pheromones. The fluorescence ligand-binding assays revealed highest affinities for hexadecanal, farnesol, and limonene with K_D_ values of 0.55 ± 0.08, 0.35 ± 0.04, and 1.54 ± 0.39, respectively. The binding sites of GOBPs from yellow peach moth were predicted using homology modeling and characterized using molecular docking approaches. The results indicated the best binding affinity of both GOBP1 and GOBP2 for farnesol, with scores of −7.4 and −8.5 kcal/mol. Thus, GOBPs may play an important role in the process of finding host plants.

## 1. Introduction

Sensing volatile chemical compounds is crucial for insects to navigate in their environment and to recognize congener. The detection of appropriate compounds and the primary sensory reactions takes place in hair-like structures of the antennae, called sensilla. The sensilla cavity is filled with fluid bathing the dendritic processes of the olfactory sensory neurons. The transfer of hydrophobic odorant molecules through the aqueous sensillum lymph towards the sensory dendritic membrane is supposed to be mediated by odorant binding proteins (OBPs) [1,2]. OBPs are small, water-soluble proteins in the sensilla lymph and depending on the structure, different types of OBPs have been identified, including pheromone binding proteins (PBPs), general odorant binding proteins (GOBPs), and antennal binding proteins (ABPs) [3,4,5,6]. So far, only two GOBP subtypes (GOBP1 and GOBP2) have been identified in lepidopteran species [7]. In previous studies it has been demonstrated that GOBP1 and GOBP2 were highly expressed in antennae and it has been suggested that they are involved in sensing host plants and oviposition sites [8,9]. More recent studies demonstrated that GOBPs also bind sex pheromones [10]. The results of Khuhro et al. [11] indicate high binding affinity of GOBPs for both host plants and the sex pheromones (Z9-16:Ald, Z11-16:Ald and Z13-18:Ald) from *Chilo suppressalis* and also a general odorant binding protein from *Sitotroga cerealella* showed high binding affinity for the pheromone HDA (7Z, 11E-hexadecadien-1-ol acetate) [12]. These studies suggested the GOBPs may also play a role in pheromone reception.

Growth conditions and sensory stimuli can cause physiological changes in a wide range of insects [13,14], including changes in the chemosensory responsiveness. It has been observed that in flies (*Drosophila melanogaster*) the responses to plant odors are depending on age [15]; similarly, in female moth (*Manduca sexta*) the response to plant odorants became more intense with increasing age and was altered upon mating [16]. Moreover, there is recent evidence that exposure to semiochemicals can elicit changes in the expression levels of distinct OBP subtypes [17]. Since the responsiveness of the olfactory system is primarily based on an interplay of different families of antennal proteins [18], it will be of fundamental interest to elucidate the structural and functional features of the molecular elements.

The yellow peach moth, *Conogethes punctiferalis* Guenée (Lepidoptera: Crambidae), mainly occurs in tropical and subtropical countries [19]. The distribution of yellow peach moth extends from Asia to Australia [20]. It is a polyphagous insect pest causing severe crop losses owing to the direct larval damage to reproductive or economically important plant parts [21]. It attacks fruits of 30 plant species belonging to 15 families [22]. It is considered the most serious insect pest of fruits and maize crop in China [23]. The main sex pheromone compounds of yellow peach moth are (E)-10-hexadecenal (E10-16:Ald), along with the two minor components (Z)-10-hexadecenal (Z10-16:Ald) and hexadecenal (16:Ald) [24,25,26]. Field trials indicate that Z10-16:Ald and 16:Ald alone do not attract males. A blend of these compounds (two or three) was more attractive [25]. Since the first discovery of insect OBP in the male antennae of the giant moth *Antheraea polyphemus* [9], a large number of olfactory genes have now been identified in numerous insect species through molecular cloning, cDNA library sequencing and genome-wide transcriptional analyses [27,28]. With regard to molecular cloning, a large number of OBP genes have been recently identified in the antennae of the yellow peach moth. In total, 15 putative odorant binding proteins (OBPs), 46 putative odorant receptors (ORs), and 7 putative ionotropic receptors (IRs) were annotated and identified as olfactory-related genes of yellow peach moth [7]. So far, binding specificity of two PBP genes in the yellow peach moth has been investigated by Ge et al., 2018 [29]. A better understanding of the molecular mechanisms of sex pheromone perception would improve the use of pheromones to control this pest. In this study, we have evaluated the expression profiles of GOBPs in yellow peach moth and analyzed the binding properties of GOBPs by fluorescence ligand-binding assays as well as molecular docking approaches.

## 2. Materials and Methods

### 2.1. Insects

Pupae of yellow peach moth were provided by the Institute of Plant Protection (IPP), Chinese Academy of Agricultural Sciences. Female and male pupae were separately kept in plastic cages (L 31 × W 26 × H 28 cm) [30]. All the tests were conducted in a controlled environment chamber at 27 ± 1 °C, with 60–70% relative humidity (RH) and 16:8 h (L:D) photoperiod. The moths were feed with 10% honey solution [31].

### 2.2. RNA Extraction and cDNA Synthesis

Total RNA extracted from different tissues of male and female adults, tissues namely antennae, head, wing, abdomen, thorax, and legs were dissected and RNA samples were extracted using the Quick-RNA^TM^ MicroPrep Kit (ZYMO Research, Irvine, CA, USA) according to the manufacturer’s instructions. Ten adults were used from both the sexes to extract antennae and legs and other tissue mentioned above was extracted using three adults per sample. The dissected tissue sample was transferred into sterile mortar/pestle and grounded with RNA lysis buffer Then the mixture was transferred to the column placed on the collection tube and centrifuged (10,000 rpm, 30 s), the flow-through was discarded. 40 μL of DNase I reaction mix was added to the tube for trace DNA removal. RNA prep buffer was added to the column and centrifuged (10,000 rpm, 30 s), following this step a wash buffer provided in the kit was added to the column and spun down. Finally, the column was placed carefully on a RNase-free tube and 15 μL DNase/RNase-free water was added and centrifuged (10,000 rpm, 30 s), the total RNA was eluted and tubes are stored at −80 °C. The integrity of the total RNA was analyzed using 1.5% agarose gel electrophoresis [32]. The quality and concentration were analyzed on a NanoDrop 2000 spectrophotometer (Thermo Scientific, USA). RNA samples were used for cDNA synthesis at absorption ratios of A260/A280 = 1.80 − 2.10. The cDNA was synthesized using RT^TM^ All-in-One Master Mix Kit (Herogen Biotech, Shanghai, China). The first strand cDNA synthesis reaction was carried out from 1 μg of total RNA. Anchored oligo (dT) from the kit is used and cDNA was synthesized by following the manufacturer’s protocol. The final cDNA samples were stored at −20 °C until further analysis.

### 2.3. Quantitative Real-Time PCR (qRT-PCR)

Expression of GOBPs in different tissues were analyzed using qRT-PCR. SYBR green (Premix Ex Taq^TM^, Takara, Japan) method was used to evaluate the expression level of GOBP genes in different tissues (ABI 7500 Fast, Applied Biosystems, Waltham, MA, USA). The primers efficiency was tested by using 10-fold diluted cDNA samples and standard curve was generated. The C_t_ values are plotted against the Log of the cDNA dilutions, efficiency percentage and R^2^ values are within the acceptable range [33]. The details of the primers and obtained efficiency values were listed in the Supplementary Table S1. Two-step program was adopted; reaction volume was set to 20 μL. The program was designed as follow: after denatured at 95 °C for 30 s, then followed by 40 cycles of 95 °C for 5 s, 60 °C for 30 s; melting curve analysis was performed from 60 °C to 95 °C to determine the specificity of PCR products. Three independent biological replicates were maintained for all the sample and four technical replicates were performed form each biological sample. 2^−ΔΔCT^ method was used to calculate the relative expression of GOBP genes to the reference gene RP49 (accession number KX668533) [34,35].

### 2.4. Oviposition Preference Test

The oviposition preference of yellow peach moth was examined using the three hosts, peach (*Prunus persica*), sweet corn (*Zea mays* L. var. saccharate), and apple (*Malus pumila* Mil). The hosts were wrapped using wet cheesecloth and placed inside the plastic cages (one host/cage), newly emerged male and female moths (30:30) were allowed into the cage and these cages are placed inside the climate controlled chambers (one cage/chamber, 27 ± 1 °C, RH 60–70% with 16:8 h (L:D) photoperiod). Moderately ripen hosts (apple, peach) and fresh sweet corn were used, fully ripened hosts cannot withstand the chamber conditions. The test continued up to 10 days, the positions of these hosts and honey solution inside the cages were changed in order to eliminate any possible effects of environmental factors [36]. The cheesecloth was changed every day, number of eggs laid on the cheesecloth by the 30 adults were counted and accounted for the oviposition preference results. Triplicate processes were maintained through the course of the experiment.

### 2.5. Effects of Host Stimulation on the Expression Profiles of GOBP Genes

Based on the oviposition results, peach was used as a stimulant. The pupae of male and female were separated and maintained in different chambers. The newly emerged male and female adults were introduced in separate cages and maintained in separate climate-controlled chambers. Totally, 150 adults were introduced in the cages and one peach was placed per cage, the host was unchanged throughout the experiment. The same set without peach was maintained and it served as the control (non-induced). The antennae from both the sexes were excised (10 adults/sample) at 0, 1, 2, 4, 6, 8, 12, 24, 48, 72, and 96 h and RNA samples were extracted.

### 2.6. Preparation of Recombinant GOBPs

The signal peptides of GOBP1 and GOBP2 (accession number: KY130468 and KT983812) were predicted by SignalP 4.1 server [37]. Signal peptides were removed from complete ORF sequences of both genes and primers were designed, using antennal cDNA as the templates the GOBP1 and 2 were amplified and cloned using pEASY-T vector (TransGen Biotech, Beijing, China). The sense and antisense primers designed for GOBP1 and GOBP2. GOBP1 sense primers: 5′-CGGGATCCGACCACAAGATCAT-3′ and antisense primer: 5′-CCAAGCTTCTAAGTCTCCGACTG-3′ (BamH I and Hind III restriction sites are underlined); GOBP2 sense primer: 5′-AGCGAATTCGTGAAGAGCACTGCT-3′ and antisense primer: 5′-CCGAAGCTTTCAGTATCTCTCCAT-3′ (EcoR I and Hind III restriction sites are underlined). The expected sequences were purified and cloned into the bacterial expression vector pET-30a (+) digested with the same enzymes. Then the plasmid was transformed into BL21 (DE3) competent cells (TransGen, Beijing, China) and the positive clone was inoculated in a liquid LB medium (4 L/protein) induced by 1 mM isopropyl-b-d-thiogalactoside (IPTG, Coollaber Science and Technology, Beijing, China) and placed in shaking incubator at 37 °C for 6 h. The induced bacterial cells were harvested from volume of 4 L liquid LB medium and centrifuged at 4 °C for 10 min (10,000 rpm). The pellets were subjected to ultrasonication and the recombinant GOBPs protein were purified by the Ni^2+^-IDA column (His tagged) with a gradient concentration imidazole washing. The precipitate was resolved using 8 M carbamide and proteins were dialyzed in PBS buffer (pH 7.4) to remove the remaining chemical impurities from the protein. Purified protein concentrations of GOBP1 and GOBP2 was determined by the method of Bradford using BSA as standard [38] The western blot method was performed to analyze the correct expression. 10 μL of purified protein sample was electrophoresed in 12% gel (ExpressPlus^TM^, Piscataway, NJ, USA). The gel was sandwiched with a polyvinylidene fluoride (PVDF) membrane and sample in the gel was transferred to the PVDF (electrophoresis; 100 V, 200mA, 1 h). The film was washed four times using PBST (1% Tween-20 in 0.5 M Phosphate-buffered saline). 5% skim milk powder in PBST was used as blocking buffer. The PVDF membrane was incubated in primary antibody His (1:1000) and placed on shaker for overnight at 4 °C. The membrane was washed again using PBST and treated with second antibody sheep anti-rabbit antibody (1:5000) at 37 °C for 1 h. A final wash was given to the membrane and developed in ImageQuant LAS 4000 (GE, Pittsburgh, PA, USA) using western optimized HRP (horseradish peroxidase) reagent (Pierce, ELC kit, Thermoscientific, USA).

### 2.7. Fluorescence Binding Assays

Fluorescence binding assay was used to measure the affinity of the GOBP1 and GOBP2 to 2 sex pheromones and 16 volatile compounds (Table 1) [29,39]. The fluorescence intensity was recorded on a FluoroMax-4 spectrophotometer (Horiba Scientific, Piscataway, NJ, USA) at room temperature using a 1 cm light path fluorimeter quartz cuvette. The fluorescent probe N-phenyl-1-naphthylamine (1-NPN) and all the tested chemicals were dissolved in HPLC purity methanol. The final concentration was prepared 2 mM. To measure the affinity of florescent ligand 1-NPN to each protein, a 2 µM solution of the protein in 50 mM Tris-HCl, pH 7.4, was titrated with aliquots of 1 mM ligand in methanol to final concentrations of 1–8 µM. The fluorescence of 1-NPN was excited at 337 nm and emission spectra were recorded between 300 and 450 nm. The affinity of other ligands was measured in competitive binding assays, using 1-NPN as the fluorescent reporter at 2 µM concentration and different concentrations of each ligands [40]. The GraphPad Prism 5 (GraphPad Software, Inc. San Diego, CA) was used to estimate the K_1-NPN_ (K_D_ of complex protein /1-NPN) values by nonlinear regression for a unique site of binding. It was assumed that the proteins were 100% active, with a stoichiometry of 1:1 (protein:ligand) at saturation. For other competitor ligands, the dissociation constants were calculated from the corresponding IC_50_ (concentrations of ligands halving the initial fluorescence value of 1-NPN) values using Microsoft Office Excel 2010, with the formula: K_D_ = [IC_50_]/(1+[1-NPN]/K_1-NPN_). In the equation, [1-NPN] in the free concentration of 1-NPN, and K_1-NPN_ is the dissociation constant of the complex protein/1-NPN.

### 2.8. CD Spectra, 3D Modeling, and Molecular Docking Studies

The secondary structures of the GOBP1 and GOBP2 proteins were analyzed by investigating the far-UV region (190–270 nm) of CD spectra recorded by MOS 450 AF/CD (Biologic, Grenoble, France), using 1 mm quartz cuvette. The GOBP1 and GOBP2 proteins were dissolved in sodium phosphate (20 mM, pH 7.2) to a final concentration of 0.2 mg/mL. The protein samples were measured three times independently. ClustalX was used to align a multiple protein sequence alignment and drawn with ESPript (http://espript.ibcp.fr/ESPript/ESPript/) [41]. A blast search of the amino acid sequence of GOBP1 and GOBP2 was conducted against the current Protein Data Bank (PDB; http://www.rcsb.org) to find structural templates. The sequence of *Bombyx mori* GOBP2 [42] was found to be more similar to the both GOBP1 and GOBP2 of yellow peach moth and used as a template to construct the 3-D structures. Modeller v9.19 (http://salilab.org/modeller) software was used to construct the 3-D structures. The template structure and protein alignment was done utilizing Modeller v9.19 software using align2d facility of Modeller [43] and Ramachandran plot was obtained by Procheck online server for model evaluation [44]. The GOBP1 and GOBP2 modeled structure were validated using the Ramachandran plot to check the structural stability of these proteins. Compounds were selected for molecular docking based on the IC_50_ values obtained through fluorescence binding assay, the compounds with least IC_50_ value were chosen for the docking studies (E10-16: Ald, Z10-16:Ald, farnesol, hexadecanal, and limonene). The SDF (structure data file) of these compounds are retrieved from PubChem database and using Open Babel v2.4.0 the files are converted into the Mol2 format. Autodock Vina suit v 4.0 associated with Chimera v1.13 was selected for the molecular docking analysis. The configuration file was loaded with grid generation parameter (Center X = 16.50, Y = 54.0 and Z = 13.50; Size X = 16, Y = 18, Z = 20.50), exhaustiveness value =24, energy range at =3 and number of modes =100 was set in the configuration file to run multiple iterations and obtain consistent results.

## 3. Results

### 3.1. qRT-PCR

The qRT-PCR was performed to localize the GOBP1 and GOBP2 genes in different tissues (antennae, head, wing, abdomen, thorax, and legs) used in the study. The tested genes were highly expressed in the antenna of both male and female adults compared with other tissues (Supplementary Figure S1).

### 3.2. Oviposition Preference

**Figure 1:**
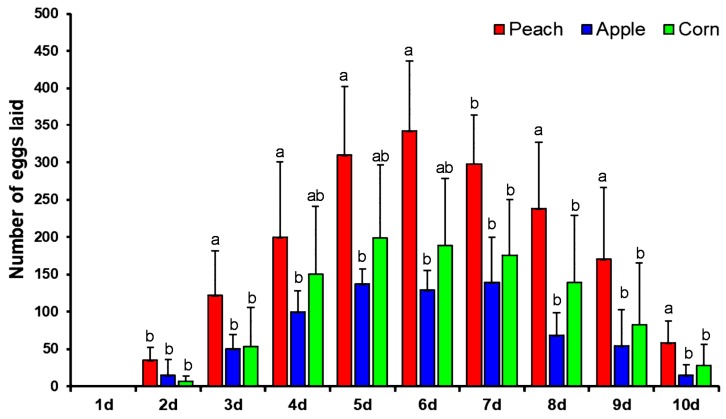
Oviposition of yellow peach moth on peach, corn and apple. Vertical bar under different letters are significantly different and same letters are not significantly different (Tukey’s, *P* < 0.05).

Females of the yellow peach moth started to oviposit on the second day after emergence with rather few eggs. In the following days, the rate of oviposition rapidly increased and peaked on days 5 to 7. The fecundity rapidly declined in following days and mortality in adults was started at day 7; a maximum number of adults died on day 10 (Figure 1). Comparing the different host plants, it becomes immediately clear that the female moths preferred to lay eggs on peach. The maximal fecundity per day was 342 eggs on peach, 198 on corn, and 139 on apple (Figure 1).

### 3.3. Expression Levels of GOBP1 and GOBP2 Genes

**Figure 2:**
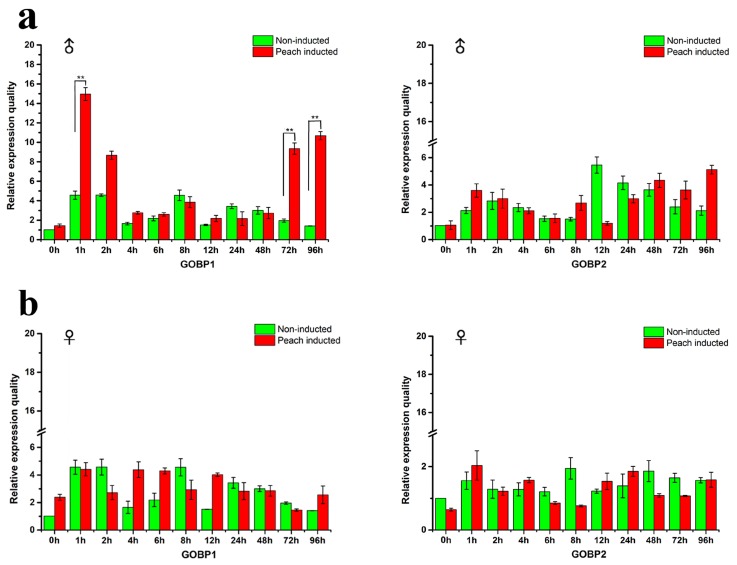
Expression profiles of *GOBP* genes in yellow peach moth antennae in different times. (**a**) and (**b**) showed the expression of *GOBP* genes in male and female moth antennae, respectively. The gene expression level between the inducted and non-inducted adults at different hours was statistically significant (*t*-test, ** *P* < 0.01).

*GOBPs* are supposed to be closely related to the reception of host volatiles. qRT-PCR experiments were performed to determine the expression level of the GOBP genes relative to the reference gene RP49 in males and females. The male and female adults were induced by the peach and compared with the non-induced individuals. The results depicted in Figure 2 indicate that upon exposure to peaches a significant increase in the expression level of *GOBP1* occurred in male antennae within the first hour (Figure 2a). Subsequently, the expression level gradually declined to the baseline. However, upon long-term exposure (72 h and 96 h) there was a significantly enhanced expression level for *GOBP1* (Figure 2b). For GOBP2 no significant changes in the expression levels were observed; neither upon short term or long term exposure to peaches. Interestingly, for females, no evidence for any significant changes in the expression levels were found.

### 3.4. Heterologous Expression and Structure Analysis GOBPs

**Figure 3:**
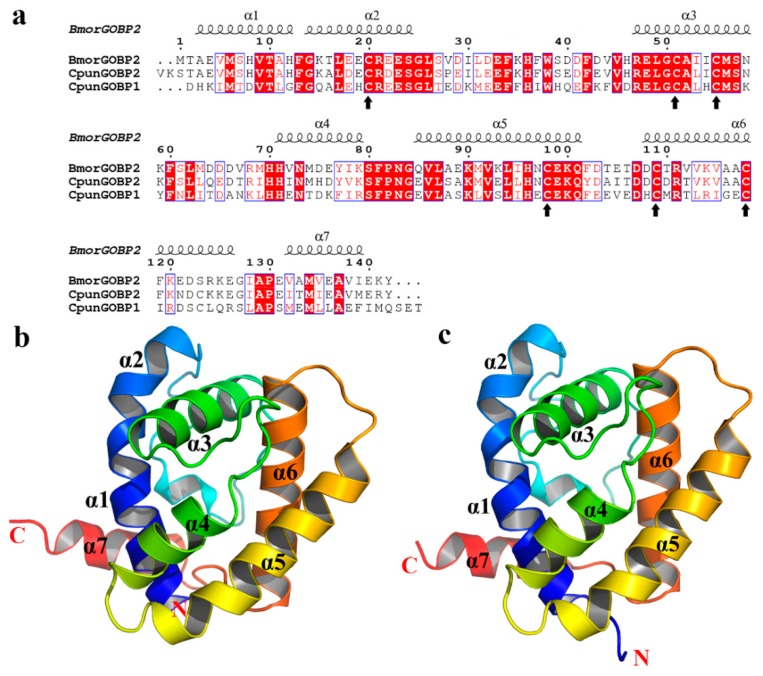
Three-dimensional models of CpunGOBP1 and CpunGOBP2: (**a**) Sequence alignment of BmorGOBP2, CpunGOBP1, and CpunGOBP2. Conserved residues are highlighted in white letters with a red background and upward arrow represents the conserved Cysteine residues in the GOBP1 and two sequences (**b**) and (**c**) were structural model of CpunGOBP1 and CpunGOBP2, respectively.

The GOBPs proteins were expressed successfully in an inclusion bodies expression system (Supplementary Figure S2). The estimated protein concentrations were 1.0 (GOBP1) and 0.8 (GOBP2) mg/mL. The molecular weight of the heterologously expressed GOBP1 and GOBP2 was determined as about 22 kDa. In order to explore the secondary structures of GOBPs, CD spectroscopy analyses were performed. The results indicated that GOBP1 and GOBP2 shared several features in their secondary structure. Similar CD spectra at three regions were found at 197, 205, and 224 nm; these regions may comprise folds with major α-helical secondary structures (Supplementary Figure S3). Structural models of the GOBPs from yellow peach moth were built based on the crystal structure of BmorGOBP2 from *Bombyx mori* [42]. The two GOBPs, CpunGOBP1 and CpunGOBP2 shared 48% and 74% identity, respectively (Figure 3a). Both predicted 3D structures of the GOBPs consisted of seven α-helices with similar amino acid sequences in this region (Figure 3b, c). Totally, eight and seven cysteines were identified in GOBP1 and GOBP2, these disulphide bonds are predicted at three positions in both proteins. In GOBP1, the Cysteines1_2 position was predicted between 119–139, 12–41, 72–130 and in GOBP2 117–137, 39–70, 74–128.

### 3.5. Flourescent Ligand-Binding Assays

In order to explore the binding characteristics of the GOBPs titration experiments were performed using N-phenyl-1-naphthylamine as fluorescent ligand. Incubating GOBPs with increasing concentrations of N-phenyl-1-naphthylamine (1-NPN) allowed to determine the affinity of both GOBPs for 1-NPN. Scatchard plot analyses of the data revealed dissociation constants of 0.74 ± 0.41μM and 0.70 ± 0.38μM for GOBP1 and GOBP2, respectively (Supplementary Figure S4).

**Figure 4:**
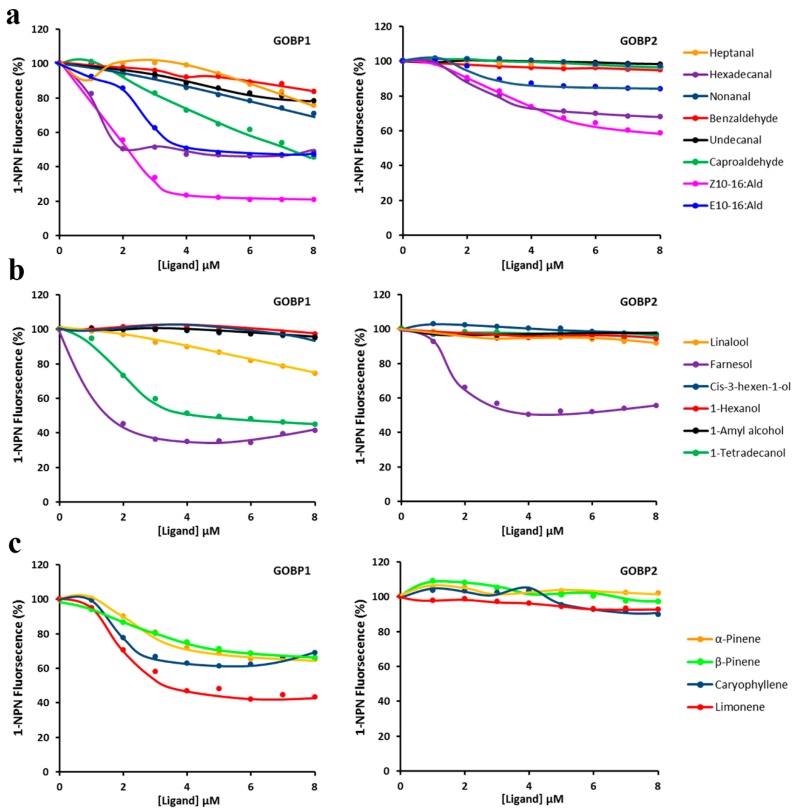
Competitive binding curves of GOBP1 and GOBP2 to different ligands. (**a**) GOBP1 and GOBP2 vs. aldehydes; (**b**) GOBP1 and GOBP2 vs. alcohols; (**c**) GOBP1 and GOBP2 vs. olefins.

To estimate the binding affinities of GOBP1 and GOBP2 for a variety of different ligands, the decrease of 1-NPN fluorescence monitored, which resulted from the ability of various compounds to displace 1-NPN (Figure 4); from the resulting data the IC_50_ and K_D_ values for the different compounds were determined (Table 1). From the 18 analyzed compounds both GOBPs showed the highest affinity for farnesol; a K_D_ value of 0.35 ± 0.04 μM was determined for GOBP1 and a value of 1.61 ± 0.03 μM for GOBP2 (Table 1). Besides, GOBP1 showed relative high affinity for some semiochemicals, most notably, hexadecanal K_D_ 0.55 ± 0.08 and limonene K_D_ 1.54 ± 0.39. In addition, some pheromonal compounds (Z10-16:Ald; E10-16:Ald) showed a reasonable binding affinity to GOBPs (Table 1).

### 3.6. Molecular Docking Studies

Based on the nucleotide sequence of GOBP1 and GOBP2 a homology modeling was performed using the Protein Databank (PDB) and the Swiss Model server to search for the perfect template which possesses the right configuration of structural conformation in each atom. GOBP1 and GOBP2 from *Bombyx mori* (PDB ID-2WCK, 2WC5) were used as template proteins for the construction of the structure. GOBP1 and GOBP2 from yellow peach moth shared 88.30% and 83.22% similarity with template proteins, respectively. Totally, 94.20 and 93.70% of amino acid residues were viewed in the core regions (most favored region) of Ramachandran plot for GOBP1 and GOBP2 modeled files. Remaining 5.85 and 6.30% of amino acid residues were found in additional or generously allowed regions. No residues found in disallowed region.

**Figure 5:**
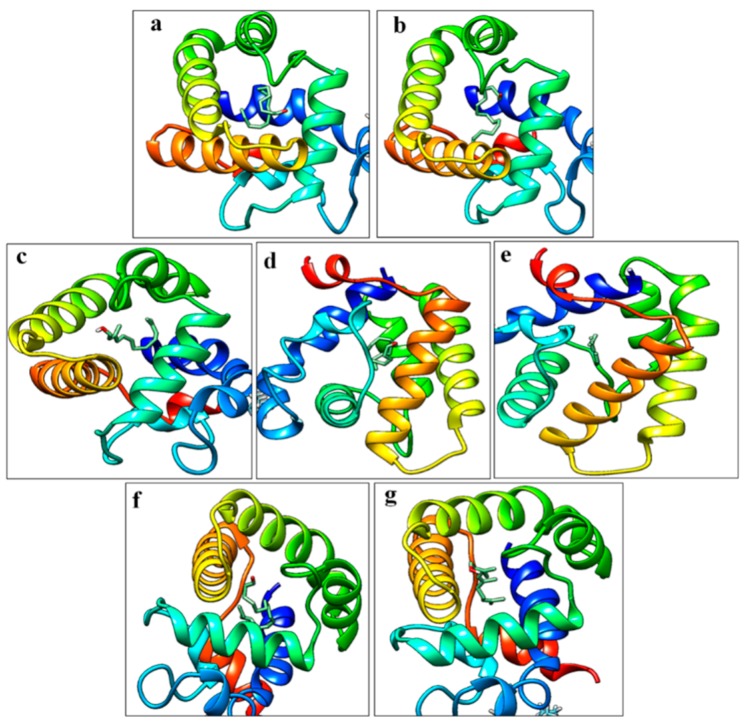
Molecular docking pose of GOBP1 and GOBP2 vs. sex pheromones and volatiles. (**a**)–(**e**) E10-16:Ald, Z10-16:Ald, farnesol, hexadecanal, and limonene vs. GOBP1 protein whereas (**f**) and (**g**) are Z10-16:Ald, farnesol vs. GOBP2.

Compounds selected for molecular docking studies (E10-16:Ald, Z10-16:Ald, farnesol, hexadecanal, and limonene) were analyzed which showed the highest affinity in the fluorescent binding assays. For GOBP1 the resulting docking scores were for hexadecanal, farnesol, and limonene −5.9, −7.4, and −6.6 kcal/mol, respectively and for Z10-16:Ald and E10-16:Ald, −5.9 and −6.2, kcal/mol. For GOBP2 the docking score for farnesol was −8.5 kcal/mol and for Z10-16:Ald −6.9 kcal/mol (Figure 5).

## 4. Discussion

Insects rely on olfaction to recognize chemical signals, which are important in controlling their behavior, including essential processes, such as mate choice and host finding. The process of odorant recognition begins when the chemical compounds encounter the odorant binding proteins in the sensillum lymph. GOBPs are considered to be involved in transferring regular odorants towards the sensory neurons. Towards an understanding of the molecular mechanisms of olfaction in the yellow peach moth, this study was concentrating on the GOBPs of this species. In order to identify relevant odorants for this species, it was found in oviposition tests that female yellow peach moth preferred the smell from peaches when searching for an oviposition site. In some species, the females prefer host plants based on the concentration of particular chemical compounds, whereas in other species the response rely on the relative proportions of volatiles emitted from plants [45,46,47]. There is also evidence that the yellow peach moth exhibits some preference for host plants based on the effects of caterpillars’ performance [48]. Further studies are needed to elucidate the parameters, which determine the host choice of yellow peach moth.

While plants often emit a large variety of volatiles, in most cases only few distinct compounds are responsible for attracting insect [49,50]. Moreover, significant changes in the responsiveness of insects to volatiles have been observed, although the underlying mechanisms are mostly unknown [51,52]. In this study, different tissues of yellow peach moth were tested firstly, and the genes were highly expressed in the antenna of both male and female adults, similar with previous studies [12,42]. Therefore, the antenna was selected for the following studies in this paper (Supplementary Figure S1). The exposure of the moth to volatiles from peaches elicited an increased expression rate of GOBP1 in males during the first hour of exposure (Figure 2a) as well as after a few days (Figure 2b). The gene expression studies showed the expression of GOBP1 in male at different hours, particularly increased expression was observed at first hour of induction and gradually decrease and again increase in expression was observed at 72^nd^ and 96^th^ hours. We postulate two possible reason for the fluctuation, on introducing the peach to the newly emerged male moths stimulated at the first hour, later the adults were adapted to the environment and exhibited decrease in expression. We also noticed the expression was triggered at 72^nd^ and 96^th^ hours, this change might cause by the host due to host remained unchanged (no fresh host provided) in the climate-controlled chamber, possible aroma released from the host might stimulated the GOBP1 once again. Another possible effect was inferred from the previous literatures, virgin male moth *Vitacea polistiformis* exhibited four-fold higher electroantennogram responses to pheromones than mated male [53]. Eager for mating also a potential reason for the male adults to have timely fluctuation in GOBP1 gene expression. In opposite, *Plutella xylostella* female adult’s response towards some green leaf volatiles was stronger than the unmated females [54]. The data obtained in our oviposition assay connects the qRT-PCR results, although we did not check the expression pattern of GOBP1 after mating. The expression of GOBP1 in female might related to the after-mating process, where the mated female adults seeks host to lay the eggs. The exact underlying mechanisms are poorly understanding. However, an elevated expression levels of distinct OBP subtypes upon exposure to semiochemicals has recently been reported by Paula et al. (2018) for the boll weevil, *Anthonomus grandis* Boheman [17]. A similar result was not observed for females, which may indicate a sex specific reaction to some of the compounds in the volatile blend emitted from the peaches.

The secondary structure of GOBP is important for the protein-ligand interactions and the disulphide bridges are supposed to play a vital role in providing the structural integrity. The CD spectroscopic study revealed the overall secondary structure of the GOBPs and allowed to predict a few folds with major α-helical secondary structures (Figure 3). The structure of insect OBPs is characterized by its compact structure with seven α-helix, thus forming a potential binding pocket for interacting volatile compounds [40,55]. GOBP1 and GOBP2 possess eight respectively seven cysteines which form three disulphide bridges; the prediction suggested interactions for GOBP1: 119–139, 12–41, and 72–130; for GOBP2: 117–137, 39–70, and 74–128. Similar results concerning the structure and the importance of the disulphide bridges have been reported for the binding proteins from *Bombyx mori* [56,57]. Analyzing the CD spectroscopic results facilitated to obtain good 3D models of GOBP1 and GOBP2. Thus, 3D modelling aided us to visualize and study the protein-ligand interactions.

In order to explore the protein/ligand interaction the binding studies using a displacement approach with a fluorescent ligand were extended by molecular docking experiments employing the Autodock Vina tool. This approach focuses on the total negative force that acts on ligands; it includes calculation of position, orientation, and torsions angle [58]. The binding energy between protein and ligands tended to be in a similar range. However, when comparing the different volatiles, it turned out that farnesol showed the highest binding score with GOBP1 and GOBP2, with values of −7.4 and −8.5 kcal/mol. In this context it is interesting to note, also that both GOBPs from the rice striped stem borer *Chilo suppressalis* [11] and *Orthaga achatina* [59] showed the highest binding affinities for farnesol. The GOBPs affinity to farnesol is more interesting, the farnesol was reported as a constituent of sex pheromone in some insects [60,61,62]. The interactions with GOBPs provides major insight (1) These GOBPs might have dual functions like recognizing the host or sex pheromones; (2) Recognizing other insects sex pheromones would help them to avoid their predators, further studies are required to analyze these sex phenomena.

There are evolutionary facts were put forth that the GOBPs are evolved from PBP by gene duplication, PBP and GOBP2 in *Manduca sexta* have close relationship and play an important role in coordinated olfactory behaviors [63,64]. Previous research published in *Bombyx mori* [41], *Spodoptera litura* [65], and *Dendrolimus tabulaeformis* [66] olfaction system states that the binding affinity of GOBP2 with sex pheromones was high as PBPs. More interestingly in our study, the data obtained from fluorescence binding assays indicated that GOBP1 from yellow peach moth can interact with both tested volatile compounds as well as the sex pheromones (Z10-16:Ald and E10-16:Ald), while GOBP2 did not exhibited the binding affinity. Similarly, GOBP1 from oriental fruit moth *Grapholita molesta* showed interaction with both host volatiles and sex pheromones, this effect inferred as GOBP1 might have dual function in recognizing both host plant volatiles and sex pheromone components, while GOBP2 seems to be mainly tuned to interact with the minor sex pheromone component like dodecanol [67]. Vice versa, PBP2 and PBP5 from yellow peach moth can bind sex pheromones with higher affinities and also could interact with some odorants [29]. In our research, we find the GOBP1 can interact potentially with sex pheromones and other volatiles tested. Overall results indicated that GOBP1 plays a key role in yellow peach moth and might possess dual functions. Our results are exactly opposite to Huang’s findings, this may indicate that GOBPs genes between different species may differ in function. Further intensive molecular techniques like gene knockdown studies are needed to prove the functions of the GOBPs.

## 5. Conclusions

We have evaluated the expression of the two GOBP types from the yellow peach moth and have studied the secondary and tertiary structure of these proteins. The more detailed information about the molecular elements of the olfactory system together with the available chemical database and ecological knowledge will contribute to elucidate the principles and mechanisms underlying the chemical communication of the yellow peach moth. The overall results of this study imply that GOBP1 may play a prominent role in the chemosensory processes involved in tracking the host and mating partners. Although the specific functional role of insect GOBPs is still elusive, detailed analyses of their interaction with relevant plant odorants, as exemplified in this study by fluorescence binding assays and molecular docking studies, will contribute in the search for bioactive chemicals. Recent advance in computational biology will help to identify or design bioactive compounds that may allow to specifically interfere with chemical communication of pest insects. Such approaches may eventually lead to more insect-specific and environmentally-friendly control agents.

## Figures and Tables

**Table 1:** IC_50_ values (µM) and dissociation constants (K_D_) (µM) of GOBP1 and GOBP2 to different ligands at pH = 7.4

Ligand	GOBP1			GOBP2		
	Intensity (%)	IC_50_ (μM)	K_D_ (μM)	Intensity (%)	IC_50_ (μM)	K_D_ (μM)
ALDEHYDES						
Heptanal	75.78 ± 5.17	13.52 ± 3.22	5.76 ± 0.64	96.80 ± 1.33	55.95 ± 1.54	23.12 ± 4.68
Hexadecanal	48.64 ± 4.35	1.29 ± 0.14	0.55 ± 0.08	67.94 ± 1.79	21.06 ± 1.83	8.70 ± 0.74
Nonanal	71.04 ± 8.80	13.35 ± 2.41	5.69 ± 0.36	96.99 ± 2.63	92.79 ± 3.83	38.34 ± 1.09
Benzaldehyde	83.58 ± 6.61	20.62 ± 1.31	8.79 ± 0.59	94.76 ± 7.32	77.12 ± 1.40	31.86 ± 3.03
Undecanal	78.33 ± 2.89	16.81 ± 2.41	7.17 ± 0.67	98.12 ± 1.92	96.67 ± 2.41	39.94 ± 9.70
n-Hexanal	45.50 ± 4.58	7.31 ± 1.43	3.11 ± 0.71	96.44 ± 1.50	68.07 ± 3.70	28.13 ± 0.62
Z10-16:Ald	20.88 ± 0.48	2.82 ± 0.44	1.20 ± 0.09	58.63 ± 2.72	8.65 ± 1.87	3.58 ± 0.62
E10-16:Ald	47.29 ± 7.52	4.90 ± 0.54	2.09 ± 0.45	84.05 ± 2.25	21.44 ± 4.15	8.86 ± 0.93
ALCOHOLS						
Linalool	74.52 ± 2.42	15.71 ± 1.61	6.70 ± 0.69	91.86 ± 0.71	44.85 ± 1.16	18.53 ± 0.48
Farnesol	41.45 ± 2.51	0.83 ± 0.09	0.35 ± 0.04	55.64 ± 1.78	3.98 ± 0.36	1.64 ± 0.03
Cis-3-hexen-1-ol	94.99 ± 1.78	78.63 ± 0.23	33.53 ± 1.21	96.93 ± 0.99	51.33 ± 1.60	21.21 ± 1.14
n-Hexanol	97.23 ± 0.89	60.35 ± 2.44	25.73 ± 0.43	94.24 ± 1.82	95.19 ± 1.68	43.88 ± 2.41
1-Amyl alcohol	95.2 ± 1.12	33.50 ± 1.02	14.28 ± 1.74	96.54 ± 1.39	98.67 ± 2.30	75.89 ± 1.39
1-Tetradecanol	45.8 ± 0.56	4.81 ± 0.72	2.05 ± 0.43	85.52 ± 1.47	54.02 ± 2.56	22.32 ± 1.89
OLEFINES						
α-Pinene	64.96 ± 3.26	11.10 ± 3.47	4.73 ± 0.48	98.57 ± 1.13	67.95 ± 4.66	28.07 ± 4.06
β-Pinene	66.21 ± 2.96	9.94 ± 2.72	4.24 ± 0.69	97.15 ± 3.95	33.70 ± 3.29	13.92 ± 1.36
Caryophllene	68.98 ± 3.16	9.09 ± 2.42	3.88 ± 0.91	89.93 ± 2.15	20.98 ± 2.10	8.67 ± 1.12
Limonene	43.27 ± 2.47	3.62 ± 0.89	1.54 ± 0.39	92.62 ± 1.21	35.47 ± 1.14	14.65 ± 2.78

The results are based on mean ± SD, through the experiment triplicates were maintained.

## Supplementary Materials

The following are available online at https://www.mdpi.com/2075-4450/10/9/302/s1; Figure S1: Tissue expression patterns of *GOBPs* in yellow peach moth quantified by qRT-PCR; Figure S2: SDS-PAGE and Western blot analysis of recombinant GOBP1 and GOBP2. a and b) SDS-PAGE analysis of GOBP1 and GOBP2; M: Protein molecular weight marker; Lane 1: bacterial pellet after sonication; Lane 2: Protein in eluent; Lane 3: Purified protein. c and d) Western blot analysis of purified GOBP1 and GOBP2; Figure S3: CD spectroscopy of GOBP1 and GOBP2; Figure S4: The binding curves and relative Scatchard plots of 1-NPN with GOBP1 and GOBP2; Table S1: Primers for *GOBP* genes quantification by qRT-PCR.

## References

[1] Kaissling K.E. (1998). Pheromone deactivation catalyzed by receptor molecules: A quantitative kinetic model. Chem. Senses.

[2] Krieger J., Breer H. (1999). Olfactory reception in invertebrates. Science.

[3] Callahan F.E., Vogt R.G., Tucker M.L., Dickens J.C., Mattoo A.K. (2000). High level expression of “male specific” pheromone binding proteins (PBPs) in the antennae of female noctuiid moths. Insect Biochem. Mol. Biol..

[4] Calvello M., Brandazza A., Navarrini A., Dani F.R., Turillazzi S., Felicioli A., Pelosi P. (2005). Expression of odorant-binding proteins and chemosensory proteins in some Hymenoptera. Insect Biochem. Mol. Biol..

[5] Zhou J.J., Kan Y., Antoniw J., Pickett J.A., Field L.M. (2006). Genome and EST analyses and expression of a gene family with putative functions in insect chemoreception. Chem. Senses..

[6] Krieger J., Gänssle H., Raming K., Breer H. (1993). Odorant binding proteins of *Heliothis virescens*. Insect Biochem. Mol. Biol..

[7] Jia X.J., Wang H.X., Yan Z.G., Zhang M.Z., Wei C.H., Qin X.C., Ji W.R., Du Y.L. (2016). Antennal transcriptome and differential expression of olfactory genes in the yellow peach moth, *Conogethes punctiferalis* (Lepidoptera: Crambidae). Sci. Rep..

[8] Vogt R.G., Rybczynski R., Lerner M.R. (1991). Molecular cloning and sequencing of general odorant-binding proteins GOBP1 and GOBP2 from the tobacco hawk moth *Manduca sexta*: Comparisons with other insect OBPs and their signal peptides. J. Neurosci..

[9] Laue M., Steinbrecht R.A., Ziegelberger G. (1994). Immunocytochemical localization of general odorant-binding protein in olfactory sensilla of the silkmoth *Antheraea polyphemus*. Naturwissenschaften.

[10] Liu N.Y., Yang F., Yang K., He P., Niu X.H., Xu W., Anderson A., Dong S.L. (2014). Two subclasses of odorant-binding proteins in *Spodoptera exiguadisplay* structural conservation and functional divergence. Insect Mol. Biol..

[11] Khuhro S.A., Liao H., Dong X.T., Yu Q., Yuan Q., Dong S.L. (2017). Two general odorant binding proteins display high bindings to both host plant volatiles and sex pheromones in a pyralid moth *Chilo suppressalis* (Lepidoptera: Pyralidae). J. Asia-Pac. Entomol..

[12] Ma M., Chang M.M., Lei C.L., Yang F.L. (2016). A garlic substance disrupts odorant-binding protein recognition of insect pheromones released from adults of the angoumois grain moth, *Sitotroga cerealella* (Lepidoptera: Gelechiidae). Insect Mol. Biol..

[13] Kubli E. (2003). Sex-peptides: Seminal peptides of the *Drosophila* male. Cell. Mol. Life Sci..

[14] Ribeiro C., Dickson B.J. (2010). Sex peptide receptor and neuronal TOR/S6K signaling modulate nutrient balancing in *Drosophila*. Curr. Biol..

[15] Devaud J.M., Acebes A., Ramaswami M., Ferrús A. (2003). Structural and functional changes in the olfactory pathway of adult *Drosophila* take place at a critical age. J. Neurobiol..

[16] Mechaber W.L., Capaldo C.T., Hildebrand J.G. (2002). Behavioral responses of adult female tobacco hornworms, *Manduca sexta*, to hostplant volatiles change with age and mating status. J. Insect. Sci..

[17] Paula D.P., Togawa R.C., do Carmo Costa M.M., Grynberg P., Martins N.F., Andow D.A. (2018). Systemic and sex-biased regulation of OBP expression under semiochemical stimuli. Sci. Rep..

[18] Jacquin-Joly E., Legeai F., Montagné N., Monsempes C., François M.C., Poulain L., Gavory F., Larsson M.C. (2012). Candidate chemosensory genes in female antennae of the noctuid moth *Spodoptera littoralis*. Int. J. Biol. Sci..

[19] Pena J., Nadel H., Pereira M., Smith D., Peña J., Sharp J., Wysoki M. (2002). Tropical Fruit pests and pollinators: Biology, economic importance, natural enemies, and control. Pollinators and pests of Annona species.

[20] CABI (2011). *Conogethes punctiferalis* datasheet. Crop Protection Compendium.

[21] Chakravarthy A., Vasudevkammar K.K. (2015). Monograph on shoot and fruit borer, *Conogethes punctiferalis* and allied species. Consortia Research Platform on Borers.

[22] Konno Y., Honda N., Matsumoto Y. (1981). Mechanism of reproductive isolation between the fruit feeding and pin ace feeding type of yellow peach moth, *Dichocrocis punctiferalis*. Jpn. J. Appl. Entomol. Zool..

[23] CPCI Crop Protection Compendium on Internet accessed in 2005.

[24] Konno Y., Arai K., Sekiguchi K., Matsumoto Y. (1982). (E)-10-Hexadecenal, a sex pheromone component of the yellow peach moth, *Dichocrocis punctiferalis* Guenée (Lepidoptera: Pyralidae). Appl. Entomol. Zoolog..

[25] Liu M., Tian Y., Li Y. (1994). Identification of minor components of the sex pheromone of yellow peach moth *Dichocrocis punctiferalis* Guenée and field trials. Insect Sci..

[26] Kyungsaeng B., Park K.C. (2005). Insect semiochemical research in Korea: Overview and prospects. Appl. Entomol. Zool..

[27] Rützler M., Zwiebel L. (2005). Molecular biology of insect olfaction: Recent progress and conceptual models. J. Comp. Physiol. A.

[28] Menini A. (2010). The Neurobiology of Olfaction.

[29] Ge X., Ahmed T., Zhang T., Wang Z., He K., Bai S. (2018). Binding specificity of two PBPs in the yellow peach moth *Conogethes punctiferalis* (Guenée). Front. Physiol..

[30] Martin N., Moore K., Musto C.J., Linn C.E. (2016). Flight tunnel response of male European corn borer moths to cross-specific mixtures of European and Asian corn borer sex pheromones: Evidence supporting a critical stage in evolution of a new communication system. J. Chem. Ecol..

[31] Braccini C.L., Vega A.S., Aráoz M.V., Teal P.E., Cerrillo T., Zavala J.A., Fernandez P.C. (2015). Both volatiles and cuticular plant compounds determine oviposition of the willow sawfly *Nematus oligospilus* on Leaves of Salix spp. (Salicaceae). J. Chem. Ecol..

[32] Cui L., Rui C.H., Yang D.B., Wang Z.Y., Yuan H.Z. (2017). De novo transcriptome and expression profile analyses of the Asian corn borer (*Ostrinia furnacalis*) reveals relevant flubendiamide response genes. BMC Genom..

[33] Bustin S., Benes V., Garson J., Hellemans J., Huggett J., Kubista M., Mueller R., Nolan T., Pfaffl M., Shipley G. (2009). The MIQE guidelines: Minimum information for publication of quantitative Real-Time PCR experiments. Clin. Chem..

[34] Livak K.J., Schmittgen T.D. (2001). Analysis of relative gene expression data using real-time quantitative PCR and the 2 (-Delta Delta C(T)) method. Methods.

[35] Yang L., Hu X.J., Xu Z.F., He L., Xiao W. (2017). Screening of reference genes for qRT-PCR in *Conogethes punctiferails* (Lepidoptera: Crambidae). Acta Entomol. Sin..

[36] Glaser N., Frérot B., Leppik E., Monsempes C., Claire C.D., Ru B.L., Lecocq T., Calatayud P.A. (2014). Similar differentiation patterns between PBP expression levels and pheromone component ratios in two populations of *Sesamia nonagrioides*. J. Chem. Ecol..

[37] Petersen T.N., Brunak S., Heijne G., Nielsen H. (2011). SignalP 4.0: Discriminating signal peptides from transmembrane regions. Nat. Methods.

[38] Bradford M.M. (1976). A rapid and sensitive method for the quantitation of microgram quantities of protein utilizing the principle of protein-dye binding. Anal. Biochem..

[39] Liu N., Zhu J., Zhang T., Dong S. (2017). Characterization of two odorant binding proteins in *Spodoptera exigua* reveals functional conservation and difference. Comp. Biochem. Physiol. Part A Mol. Integr. Physiol..

[40] Yu Y., Ma F., Cao Y., Zhang J. (2012). Structural and functional difference of pheromone binding proteins in discriminating chemicals in the gypsy moth, *Lymantria dispar*. Int. J. Biol. Sci..

[41] Li Z.Q., Zhang S., Luo J.Y., Zhu J., Cui J.J., Dong S.L. (2015). Expression analysis and binding assays in the chemosensory protein gene family Indicate multiple roles in *Helicoverpa armigera*. J. Chem. Ecol..

[42] Zhou J.J., Robertson G., He X., Dufour S., Hooper A.M., Pickett J.A., Keep N.H., Field L.M. (2009). Characterisation of *Bombyx mori* odorant-binding proteins reveals that a general odorant-binding protein discriminates between sex pheromone components. J. Mol. Biol..

[43] Marti-Renom M., Stuart A., Fiser A., Sanchez R., Melo F., Šali A. (2000). Comparative protein structure modeling of genes and genomes. Annu. Rev. Biophys. Biomol. Struct..

[44] Laskowski R., Rullmannn J., MacArthur M., Kaptein R., Thornton J. (1996). Thornton AQUA and PROCHECK-NMR: Programs for checking the quality of protein structures solved by NMR. J. Biomol. NMR.

[45] Thompson J.N., Pellmyr O. (1991). Evolution of oviposition behavior and host preference in lepidoptera. Annu. Rev. Entomol..

[46] Bruce T.J.A., Wadhams L.J., Woodcock C.M. (2005). Insect host location: A volatile situation. Trends Plant Sci..

[47] Najar-Rodriguez A.J., Galizia C.G., Stierle J., Dorn S. (2010). Behavioral and neurophysiological responses of an insect to changing ratios of constituents in host plant-derived volatile mixtures. J. Exp. Biol..

[48] Du Y.L., Zhang J.X., Yan Z.G., Yang M.M., Zhang M.Z., Zhang Z.Y., Qin L., Cao Q. (2016). Host preference and performance of the yellow peach moth (*Conogethes punctiferalis*) on chestnut cultivars. PLoS ONE.

[49] Matsui K. (2006). Green leaf volatiles: Hydroperoxide lyase pathway of oxylipin metabolism. Curr. Opin. Plant Biol..

[50] Scala A., Allmann S., Mirabella R., Haring M.A., Schuurink R.C. (2013). Green leaf volatiles: A plant’s multifunctional weapon against herbivores and pathogens. Int. J. Mol. Sci..

[51] Gadenne C., Renou M., Sreng L. (1993). Hormonal control of pheromone responsiveness in the male black cutwormAgrotis ipsilon. Experientia.

[52] TURGEON J.J., McNEIL J.N., ROELOFSt W.L. (1983). Responsiveness of *Pseudaletia unipuncta* males to the female sex pheromone. Physiol. Entomol..

[53] Pearson G.A., Schal C. (1999). Electroantennogram responses of both sexes of grape root borer (Lepidoptera: Sesiidae) to synthetic female sex pheromone. Environ. Entomol..

[54] Reddy G.V., Guerrero A. (2000). Behavioral responses of the *Diamondback* moth, *Plutella xylostella*, to green leaf volatiles of *Brassica oleracea* Subsp. Capitata. J. Agric. Food Chem..

[55] Sandler B.H., Nikonova L., Leal W.S., Clardy J. (2000). Sexual attraction in the silkworm moth: Structure of the pheromone-binding-protein–bombykol complex. Chem. Biol..

[56] Leal W.S., Nikonova L., Peng G. (1999). Disulfide structure of the pheromone binding protein from the silkworm moth, *Bombyx mori*. FEBS Lett..

[57] Scaloni A., Monti M., Angeli S., Pelosi P. (1999). Structural analysis and disulfide-bridge pairing of two odorant-binding proteins from *bombyx mori*. Biochem. Biophys. Res. Commun..

[58] Trott O., Olson A.J. (2010). AutoDock Vina: Improving the speed and accuracy of docking with a new scoring function, efficient optimization, and multithreading. J. Comput. Chem..

[59] Liu S.J., Liu N.Y., He P., Li Z.Q., Dong S.L., Mu L.F. (2012). Molecular characterization, expression patterns, and ligand-binding properties of two odorant-binding protein genes from *Orthaga achatina* (butler) (lepidoptera: Pyralidae). Arch. Insect Biochem. Physiol..

[60] Free J., Ferguson A., Pickett J. (1981). Evaluation of the various components of the Nasonov pheromone used by clustering honeybees. Physiol. Entomol..

[61] Schiestl F., Ayasse M. (2000). Post-mating odor in females of the solitary bee, *Andrena nigroaenea* (Apoidea, Andrenidae), inhibits male mating behavior. Behav. Ecol. Sociobiol..

[62] Bergman P., Bergstrom G. (1997). Scent marking, scent origin, and species specificity in male premating behavior of two Scandinavian bumblebees. Can. Field-Naturalist..

[63] Merritt T.J., LaForest S., Prestwich G.D., Quattro J.M., Vogt R.G. (1998). Patterns of gene duplication in lepidopteran pheromone binding proteins. J. Mol. Evol..

[64] Vogt R.G., Rogers M.E., Franco M., Sun M. (2002). A comparative study of odorant binding protein genes: Differential expression of the PBP1-GOBP2 gene cluster in *Manduca sexta* (Lepidoptera) and the organization of OBP genes in *Drosophila melanogaster* (Diptera). J. Exp. Biol..

[65] Liu N.Y., Yang K., Liu Y., Xu W., Anderson A., Dong S.L. (2015). Two general-odorant binding proteins in *Spodoptera litura* are differentially tuned to sex pheromones and plant odorants. Comp. Biochem. Physiol. A Mol. Integr. Physiol..

[66] Zhang S., Zhang Z., Wang H., Kong X. (2014). Molecular characterization, expression pattern, and ligand-binding property of three odorant binding protein genes from *Dendrolimus tabulaeformis*. J. Chem. Ecol..

[67] Li G., Chen X., Li B., Zhang G., Li Y., Wu J. (2016). Binding properties of general odorant binding proteins from the oriental fruit moth, *Grapholita molesta* (Busck) (Lepidoptera: Tortricidae). PLoS ONE.

