# Supplementary material for: GOBP1 Plays a Key Role in Sex Pheromones and Plant Volatiles Recognition in Yellow Peach Moth, Conogethes punctiferalis (Lepidoptera: Crambidae)
Source: Insects. 2019 Sep 17;10(9):302. doi: 10.3390/insects10090302 (PMC6780721; doi:10.3390/insects10090302)
Supplement: Supplementary file 1 [file insects-10-00302-s001.zip › Supplementary files/Supplementary Table 1.docx]

**Table 1.** Primers for *GOBP* genes amplification quantification and primer stability analyzed by qRT-PCR.

| **Gene access number** | **Primer Name** | **Sequence (5'-3')** | **Slope** | **R^2^ value** | **Efficiency (%)** |
| --- | --- | --- | --- | --- | --- |
| KY130468 | *GOBP1*-Sense | GCACCACGAGAACACCGACAA | Y= −.257 X + 24.27 | 0.97 | 99.87% |
|  | *GOBP1*-Antisense | GAACTCCGCCAGCAGCAT |  |  |  |
| KT983812 | *GOBP2*-Sense | GCTGCCCATTTGGTTGTA | Y= −3.199 X + 24.00 | 0.97 | 105.4% |
|  | *GOBP2*-Antisense | CTCATCCCTGCATTCGTC |  |  |  |
| KX668533 | *RP49*-Sense | GCGTAACTGGCGTAAACCC | Y= −3.305 X + 21.49 | 0.99 | 100.71% |
|  | *RP49*-Antisense | GCGGACGTTGTGTACTAGTA |  |  |  |
