# Supplementary figures and images for: GOBP1 Plays a Key Role in Sex Pheromones and Plant Volatiles Recognition in Yellow Peach Moth, Conogethes punctiferalis (Lepidoptera: Crambidae)
Source: Insects. 2019 Sep 17;10(9):302. doi: 10.3390/insects10090302 (PMC6780721; doi:10.3390/insects10090302)

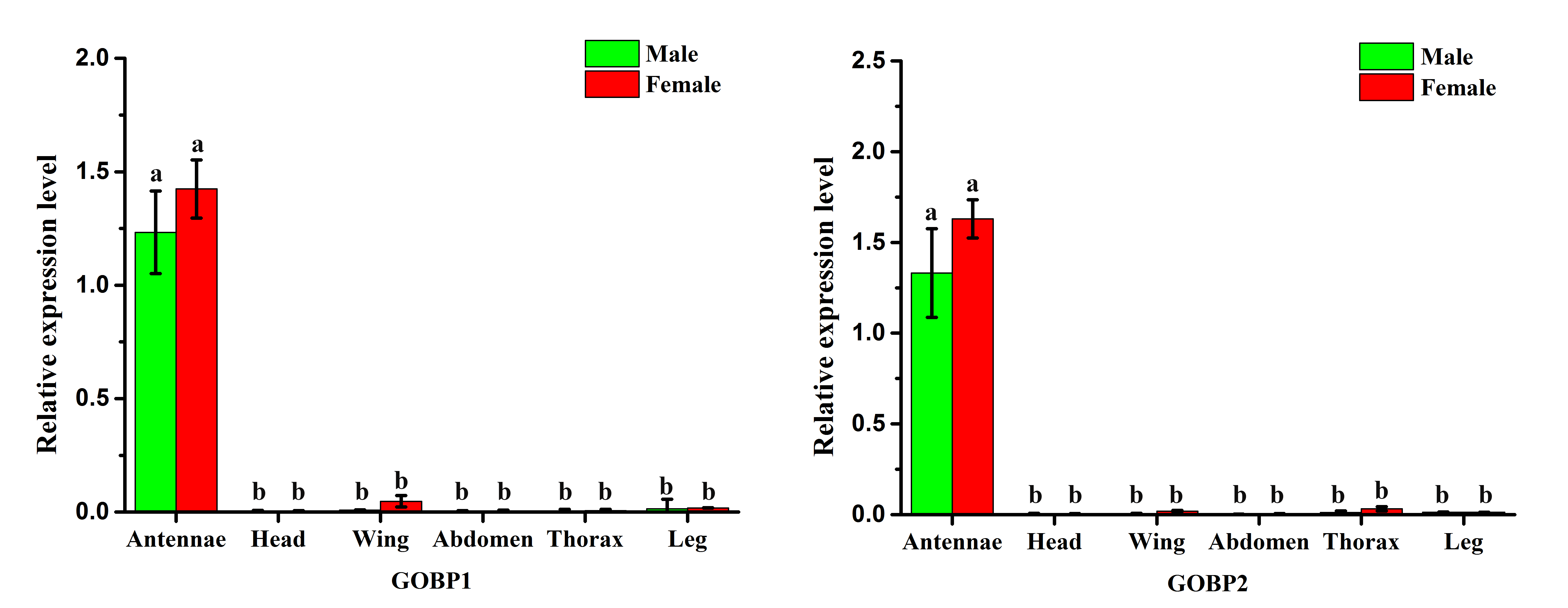

Supplement: Supplementary file 1 [file insects-10-00302-s001.zip › Supplementary files/Figure S1.tif]

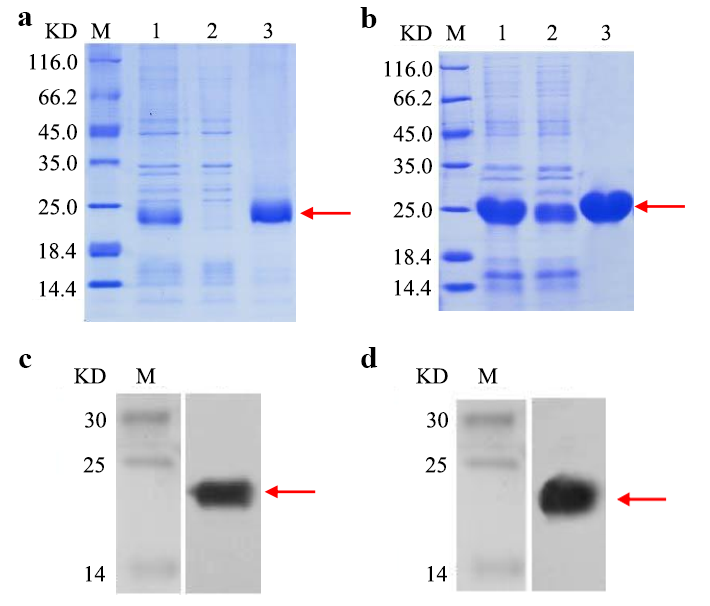

Supplement: Supplementary file 1 [file insects-10-00302-s001.zip › Supplementary files/Figure S2.tif]

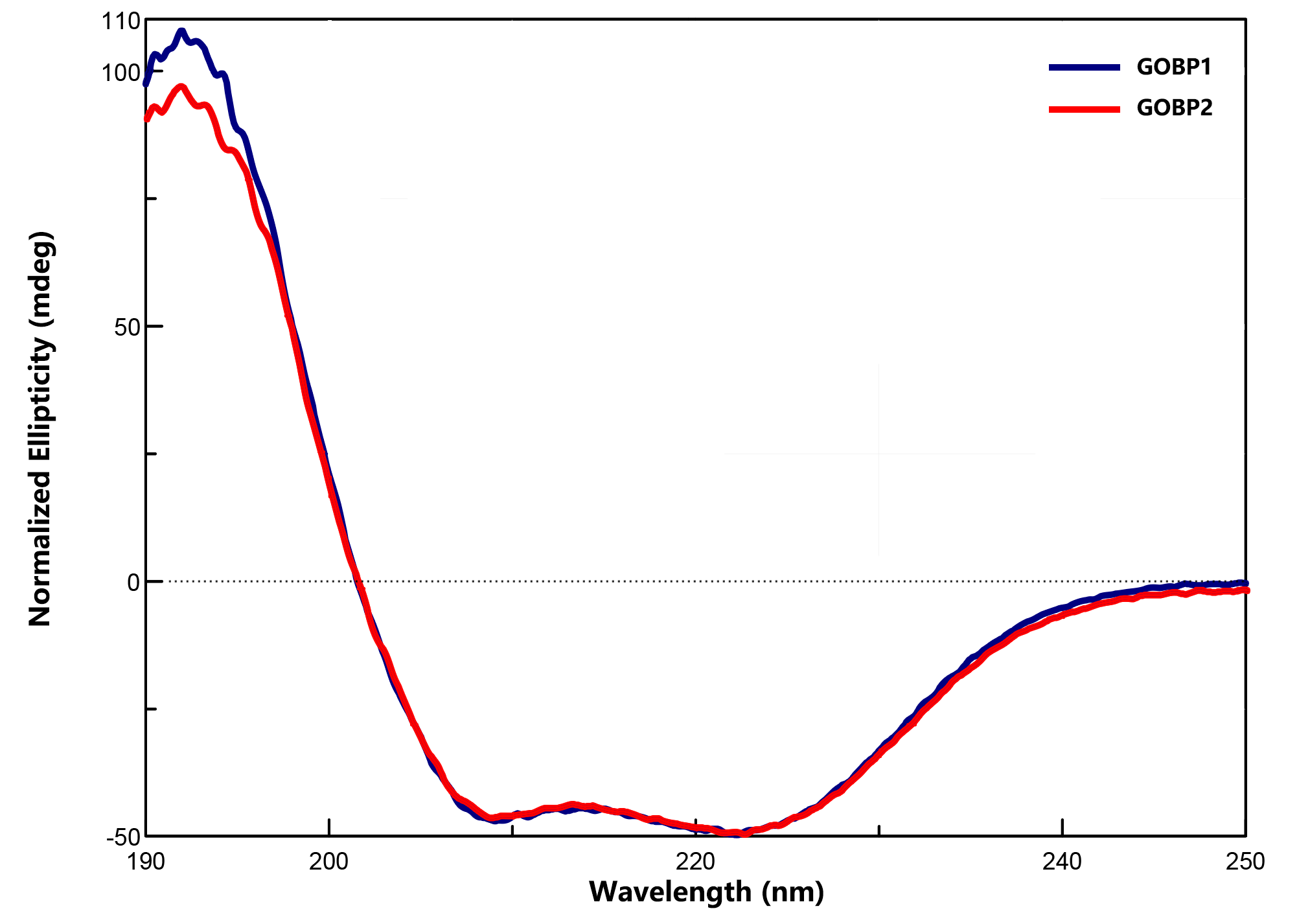

Supplement: Supplementary file 1 [file insects-10-00302-s001.zip › Supplementary files/Figure S3.tif]

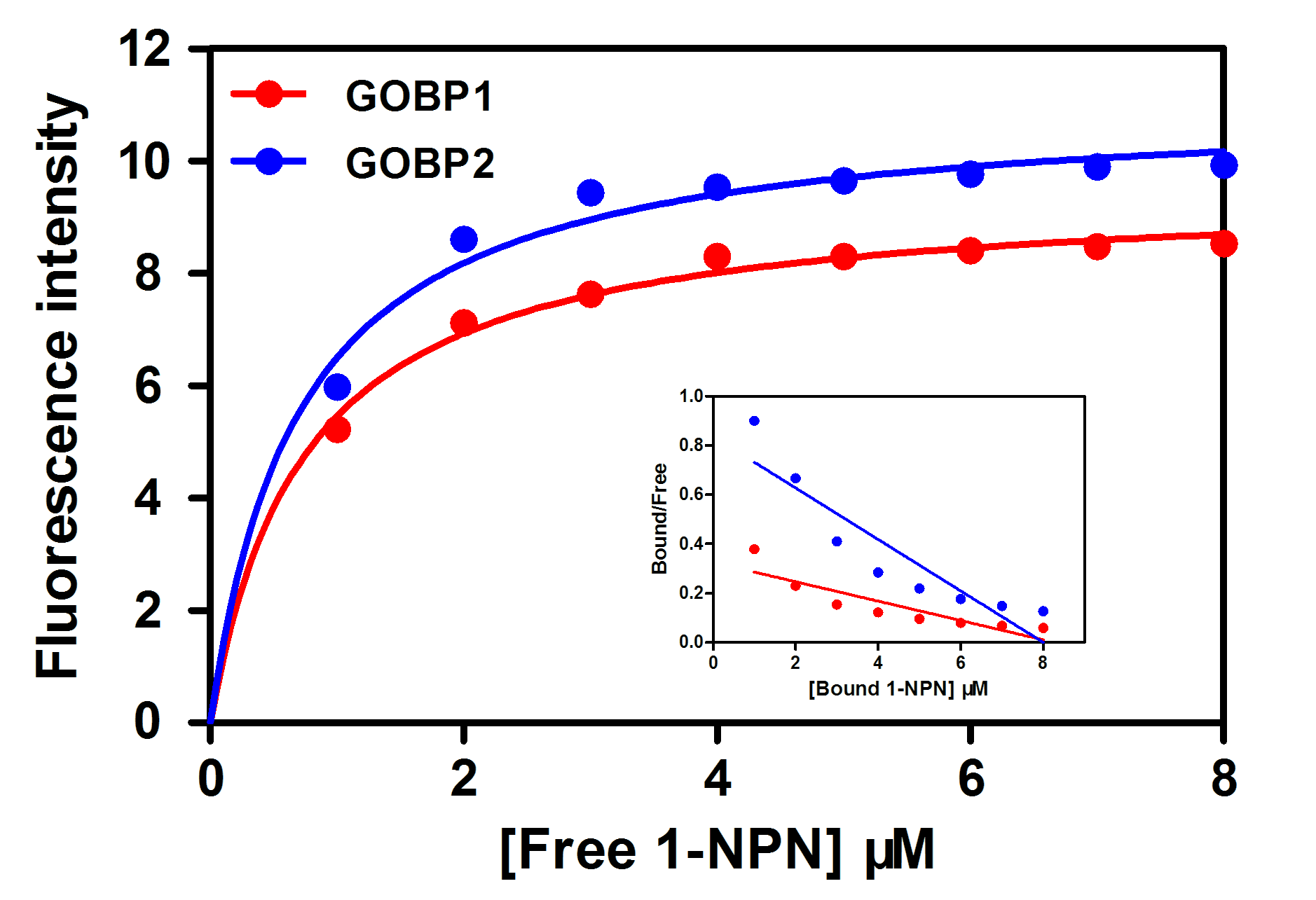

Supplement: Supplementary file 1 [file insects-10-00302-s001.zip › Supplementary files/Figure S4.tif]
